# What Contributes to the Development and Maintenance of School Refusal in Chinese Adolescents: A Qualitative Study

**DOI:** 10.3389/fpsyt.2021.782605

**Published:** 2021-12-15

**Authors:** Liang Liu, Hong Gu, Xudong Zhao, Yanbo Wang

**Affiliations:** ^1^Clinical Research Center for Mental Disorders, Shanghai Pudong New Area Mental Health Center, School of Medicine, Tongji University, Shanghai, China; ^2^Department of Psychosomatic, Shanghai East Hospital, School of Medicine, Tongji University, Shanghai, China; ^3^Division of Medical humanities and Behavioral Sciences, School of Medicine, Tongji University, Shanghai, China

**Keywords:** adolescents, school refusal, interview, interpretative phenomenological analysis, qualitative study

## Abstract

**Objective:** Although, there has been a growing number of studies on school refusal in Western cultures, the underlying factors that contribute to school refusal in Chinese adolescents remain unclear. This study aimed to better understand why Chinese adolescents refuse to go to school and to further interpret what they want to express through their school refusal behaviors.

**Methods:** We performed a qualitative study using an interpretative phenomenological analysis. Twenty adolescents with school refusal experiences were recruited from the clinical psychology department of two mental health hospitals in Shanghai, China. They participated in semistructured, face-to-face in-depth interviews. The interviews were transcribed verbatim and analyzed according to the guidelines of interpretative phenomenological analysis.

**Findings:** Five main superordinate themes emerged from data analysis: (a) competition-oriented social environment; (b) family living space dominated by conflicts; (c) personal living space lacking meaningful support; (d) conflict between the pros and cons of being labeled with a psychiatric diagnosis; and (e) reintegration in school life.

**Conclusions:** Our analysis emphasized the complex interacting effects of the social environment, family interpersonal conflicts, personal psychological factors and mental health complaints on the development and maintenance of Chinese adolescents' school refusal. These factors contributed to school refusal at each level and influenced each other's effects on school refusal behaviors. Therefore, interventions for Chinese teenagers with school refusal may need to integrate strategies that inspire reorganization and changes in different ecosystems, such as strategies related to government policy, peer relationships, family systems and individual inner dynamics.

## Introduction

School refusal behavior refers to a child's refusal to go to school and/or persistent difficulty remaining in class for the entire school day (1). A student who is reluctant or refuses to attend school usually experiences emotional distress that is temporal and indicative of aversion to attendance (e.g., excessive fearfulness, temper tantrums, unhappiness, unexplained physical symptoms) or emotional distress that is chronic and hindering attendance (e.g., depressive affect; sleep problems), usually but not necessarily manifest in absence (e.g., late arrivals; missing whole school days; missing consecutive weeks, months, or years) (2). School refusal can be a source of considerable distress for young people and their families and affects ~1% of pupils; children with school refusal behavior account for 5% of the children seeking psychiatric consultation (3). Previous investigations have indicated that boys and girls are nearly equally likely to show school refusal (4).

Adolescence is a sensitive period during which the risks of serious consequences of school refusal are highest (5). For youth, school plays an important role in their acquisition of knowledge and development of socialization skills. Sustained school absenteeism can lead to negative consequences, including poor social functioning, unemployment in adulthood, impaired social performance, and school dropout (6). Hence, research exploring the factors contributing to school refusal in adolescents can promote the design of more effective treatment, which might lead to a more positive prognosis.

One of the features of school refusal is the heterogeneity of the affected population at a causal level, that is, in terms of both the reasons for individuals' behavior and their response behavior (7). Moreover, previous research from Western culture has suggested that there are many factors contributing to school refusal and that the interaction mechanisms among these factors might be complex. Therefore, it is difficult for researchers to establish a single classification model to determine the causal relationships between school refusal and the correlated contributors based on a certain study (7). To date, the results from prior studies have suggested that the factors contributing to the development and maintenance of school refusal involve individual psychological variables, the family interpersonal environment, the institutional climate and the socioeconomic environment and culture.

### Individual Psychological Variables

Previous research has implied links between children's school refusal and various individual psychological factors, such as emotional distress, feelings of burnout, dysfunctional emotion regulation and childhood trauma. For example, Devenney and O'Toole's (8) study found that children's school refusal could be predicted by adverse childhood experience, anxiety, depression, conduct disorder, and attention deficit hyperactivity disorder. A study on 1,842 Spanish adolescents suggested that students who rejected school were at a higher risk of developing social anxiety problems (9). Meanwhile, school burnout, understood as emotional, physical and mental exhaustion due to education, was found to cause students to refuse to go to school (10). Moreover, an investigation of 184 adolescents demonstrated that children's expressive suppression was linked to their school refusal (11).

### Familial Interpersonal Environment

Prior studies have suggested that the development of school refusal is correlated with a dysfunctional family environment and parenting style (12). For example, children with school refusal behaviors reported more interpersonal conflicts and enmeshment with their parents (9, 13, 14). Meanwhile, other factors demonstrated to impact school refusal in adolescents include parental psychopathology, familial psychiatric disorders, poor family cohesion, considerable conflicts or isolation, parental overprotectiveness, ineffective parental control, separation and divorce, poverty, and parental psychological control (11, 15, 16).

### Institutional Climate

A previous research has suggested that there are correlations between school absenteeism and school factors such as interpersonal conflicts with peers, teacher-student relations and class climate (13, 14). For example, a study on Norwegian students from the 6th to 10th grades suggested that poor relationships with classmates and perceived poor support from teachers increased the risk of school refusal (6). Similarly, Hendron and Kearney (17) study revealed significant links between school absenteeism severity and poor interclassmate and student-teacher relationships.

### Socioeconomic Status and Culture

Socioeconomic status and culture are important macro factors that affect students' schooling. An investigation in Spain indicated that among students with school refusal problems, 37% were raised in economically disadvantaged environments, while only 19% came from advantageous environments (16). An investigation of 600 Indian adolescent girls indicated that school absenteeism was associated with physical pain and culture-related embarrassment during menstruation. The subjects reported that menstruation restricted their daily activities at school and that they chose to be absent from school due to the lack of privacy at school (18). Additionally, Kearney's study identified four potential conditions and causes of school refusal behavior in English-speaking areas, including *escape from aversive social and/or evaluative situations, pursuit of attention from significant others*, and *pursuit of tangible reinforcement outside of school*. The validity and reliability of this model has been proven in non-English-speaking countries such as Germany and Ecuador (19, 20). Devenney and O'Toole explored education professionals' views and experiences of school refusal within second-level schools in Ireland. Key themes included the influence of family socioeconomic status, unequal access to support services and pressures for academic achievement (8).

Additionally, socioeconomic status also affect the screening of students with school absenteeism and their access to care. For example, Martin et al.'s study showed that migrant school refusers were less likely to be identified with school refusal by school professionals, and more likely to be considered as truants. Therefore, they were less likely to be offered appropriate mental health care (21).

### Background and Aims of Current Study

Although there has been a growing number of studies on school refusal in Western culture, the underlying factors that contribute to school refusal in Chinese adolescents and the mechanism by which these potential factors interact with each other remain unclear. The impact of Chinese sociocultural factors on this phenomenon has not been investigated. In psychological and psychiatric clinics in China, children's school refusal has become the most concerning problem in need of solutions (22). Chinese culture is deeply influenced by Confucianism, and the advocacy of education has a long history (23). Due to the large population and limited qualified education resources, academic performance has become the main means of identifying educational talent and socially stratifying Chinese students. This implies that Chinese adolescents may experience greater academic stress than their Western counterparts and that long school absence may have more serious impacts on young Chinese people's mental health and occupational development (23, 24).

Hence, the aims of this study are (1) to better understand why adolescents refuse to go to school in Chinese culture and (2) to further describe what they want to express through their school refusal behaviors. Because this was an exploratory study on Chinese adolescents' subjective experiences of school refusal, a qualitative research approach was adopted to explore the participants' opinions (25). Moreover, according to the results of previous Western studies, the development of school refusal is inseparable from the intertwined effects of the social environment, the school education environment, the family environment and personal emotional disorders. This is consistent with ecological system theory (EST), which emphasizes that “individual development is nested in a series of environmental systems that influence each other” (26, 27). Thus, EST was adopted as the framework of reference for the current study.

## Methods

### Study Design and Methodology

Interpretative phenomenological analysis (IPA) was performed in this study because it has emerged as an increasingly popular methodological tool that enables the in-depth exploration of the meaning of specific issues that are pertinent to participants (28). The use of this methodology allowed us to examine the participant's subjective experiences in all their diversity and frame questions about the participants' cognitive and affective processes (29).

### Participants

Twenty adolescents (10 boys and 10 girls) aged 13–18 years old were recruited through purposive sampling between May 2020 and April 2021 from the outpatient clinical psychology departments of two mental health hospitals in Shanghai, China. Contact was made *via* psychiatrists and psychotherapists working with the adolescents. The sample size was determined based on data saturation—e.g., at the point when no new themes emerged from the participants' experiences. All the participants fulfilled Berg's criteria for school refusal: (1) an unwillingness or refusal to attend school, which often leads to excessive and prolonged school absence, by children who (2) stay home during school hours with their parents' knowledge rather than concealing the problem; (3) experience physical symptoms or emotional distress such as anxiety, depression, and unhappiness at the prospect of attending school; (4) do not show severe antisocial behavior; and (5) whose parents have made reasonable efforts to guarantee the child's safety at school (30). Meanwhile, youth with intellectual disability were excluded. Eight adolescents were also diagnosed with mental health disorders, such as major depressive disorder, bipolar disorder and generalized anxiety disorder. Five participants had been receiving medication, and 12 were accepting psychotherapy when they were enrolled in this study. The participants' demographic characteristics are listed in Table 1.

**Table 1:** Characteristics of the participants.

**IP**	**Age**	**Grade**	**Gender**	**Number of siblings**	**Birth order**	**Parents' marital status**	**Length of SR (month)**	**Psychiatric diagnosis**
P1	13	JHS	M	1	2nd	Divorced	0.5	None
P2	15	JHS	F	0	1st	Married	1	MDD
P3	12	JHS	M	0	1st	Married	15	None
P4	14	JHS	M	0	1st	Divorced	7	None
P5	16	JHS	M	1	1st	Divorced	8	None
P6	14	JHS	F	0	1st	Married	3	None
P7	17	HS	F	1	1st	Married	5	GAD
P8	18	HS	F	0	1st	Remarried	10	MDD
P9	18	HS	F	0	1st	Married	13	None
P10	15	JHS	M	0	1st	Married	9	None
P11	16	HS	M	0	1st	Married	16	None
P12	17	HS	F	0	1st	Married	5	MDD
P13	13	JHS	M	0	1st	Married	14	None
P14	14	JHS	M	0	1st	Divorced	6	None
P15	16	HS	F	0	1st	Married	10	BD
P16	18	HS	M	0	1st	Married	8	BD
P17	17	HS	F	1	1st	Married	7	MDD
P18	15	JHS	F	0	1st	Married	9	MDD
P19	17	HS	M	0	1st	Married	9	None
P20	12	JHS	F	0	1st	Married	4	None

*M, male; F, female; MDD, major depressive disorder; BD, bipolar disorder; GAD, Generalized anxiety Disorder; HS, high school; JHS, junior high school*.

### Ethical Standards

Ethics approval for this research was received from the ethics committee of Tongji University as well as the Shanghai Pudong New Area Mental Health Center (No. PWRd2020–01). Participants were informed of the purpose of the research, after which written informed consent was obtained from the adolescents and their parents for their child's participation in this study. Written informed consent was also obtained from the parents of the adolescents for the publication of their research data. Confidentiality was ensuring by using numbers instead of names (e.g., P1, P2) and removing identifying information from the transcripts.

### Data Collection

Semistructured interviews were performed with every participant in a private interview room. The interviews were conducted by three researchers who had conducted qualitative research for at least 1 year. The duration of each interview was ~60 min. With participant permission, all interviews were audio-recorded. A broad data-generating question was first asked: “Please tell me about your experiences after school refusal.” Open-ended follow-up questions were used to obtain detailed descriptions. Probing questions, such as “Please tell me more about that,” were used to enhance the depth of discussion. The guiding questions that were loosely followed in the interviews were as follows:

1. Can you talk about your experience with studying in school before? What is your opinion on the meaning of going to school?
2. How were the relationships between you and your classmates and teachers before?
3. When did you decided not to go school? Can you talk about how this idea came about?
4. What do you think would be the reasons that led to your school absence? How did you and your families make this decision at that time?
5. How did the people around you react when you stopped going to school? How did you respond to their reactions?
6. Do you have any plans for your future life?
7. Do you have any worries about going back to school in the future? Have you or people around you tried to help you back to school? If you have, what methods have you tried? Did those methods work well or not? Why?

### Data Analysis

The transcribed interviews were analyzed following Smith et al.'s (28) guidelines. The transcripts were read several times, and the left-hand margin was used to annotate what was interesting or significant about what the respondent said. The second step was initial noting, in which the semantic content and language use were examined in a very exploratory manner. The process of initial coding included descriptive, linguistic, and conceptual comments. The third step was developing emergent themes. The main task in this process was to reduce the volume of detail while maintaining complexity in terms of mapping the interrelationships, connections, and patterns among the exploratory notes. The fourth step consisted of further reducing the data by establishing connections between the preliminary themes and clustering them appropriately. Finally, a table was produced that showed each higher-order theme and the subthemes that composed them. After the analysis of each interview separately, connections among the superordinate themes across the interviews were identified.

Several strategies were used to ensure trustworthiness and credibility. Two coauthors (LL and YW) analyzed the transcripts independently by bracketing their preconceived ideas and strictly following the adapted method from Smith et al. described above. The findings were then compared and discussed by two authors until consensus on themes, theme clusters, and superordinate themes was achieved. Meanwhile, an audit trail was maintained to ensure that all analysis steps could be traced back to the original interviews. After completing the analysis, we chose three analytic texts to return to the participants and asked them to evaluate whether the text analysis was in line with their real-life experiences.

## Results

**Figure 1:**
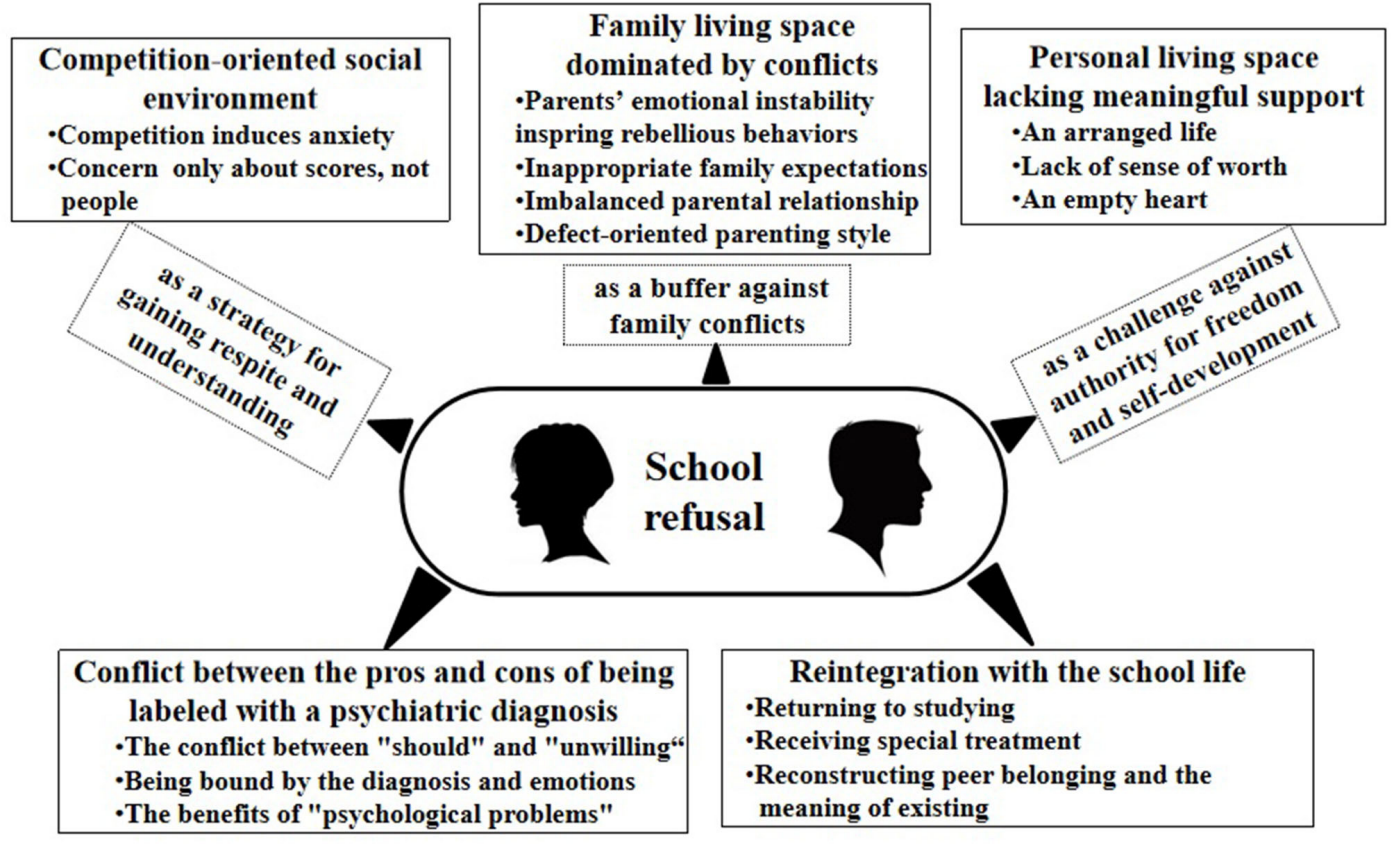
The map of the themes.

Five interconnected and relatively independent superordinate themes emerged from the data analysis: (1) competition-oriented social environment, (2) family living environment dominated by conflicts, (3) personal living space lacking meaningful support, (4) conflict between the pros and cons of being labeled with a psychiatric diagnosis, and (5) reintegration in school life. Eighteen subthemes were identified in relation to these 5 master themes. The main results are shown in Figure 1.

### Competition-Oriented Social Environment

Almost every adolescent in our study experienced pressure caused by academic competition. Adolescents' academic performance was considered the most important criterion for evaluating their excellence and for subsequent talent selection. However, the significance of other aspects of their existence was ignored to some extent.

#### Competition Induces Anxiety

Eighteen teenagers reported that they felt great pressure due to the fierce competition with their classmates in terms of scores. In daily conversations, parents and teachers often compared their children's academic performance with that of their peers. They also created an atmosphere in which the following idea was communicated: “if you get poor scores, you will be eliminated, and no one will like you.” Teenagers felt exhausted due to bearing this pressure. They became depressed, anxious and self-critical.

“My dad himself is very anxious; he always tells me that if I sleep more than 5 h a day, I'll be washed out. Because when I am sleeping, all my classmates are studying hard. School teachers also compare their classmates with each other frequently. Students who can get high scores are always popular with their teachers and classmates. This made me feel nervous, angry and helpless”—P19.

Some teenagers even said that this competitive atmosphere had existed since kindergarten. They felt deeply drained.

“Just like most of my classmates, we have been taking various training courses since kindergarten. The purpose of all these is to win the competition with my peers so we can get into good primary and middle schools and universities. I have hated going to school from the time I was little. It is very boring and tiring for me”—P15.

This kind of competitive environment also triggered anxiety among teachers. The students' scores were regarded as indicators of the teachers' work performance, which could affect teachers' career development.

“At the end of every semester, our teachers are ranked according to the scores of the students in their classes. If the students do not perform well-enough, the teacher will be replaced. So, our teachers are also under pressure. They transfer their stress to us and push us to learn much harder”—P7.

#### Concern Only About Scores, Not People

All teenagers in our study complained that their parents and teachers used test scores as the only criterion to judge their value. They felt that the adults did not care about them and their pain and did not see their value aside from their academic performance. This made them angry, stressed and frustrated.

“For my parents, scores are everything. It doesn't matter to them if I live happily or if am I having a tough time or not. They either don't care about what skills and advantages I have besides studying. They don't care about me”—P3.

“Our headteacher only cares if we can get high scores or not. Many classmates of mine became depressed due to academic pressure. Some even hurt themselves or committed suicide. However, to my surprise, he (the headteacher) said that the students who were depressed were malingering. He asked them to do more math papers to cure themselves”—P5.

Six participants reported that teachers treated students differently based on their test scores, which they perceived as unfair and which made them feel helpless.

“Our teachers treat the students unfairly. It doesn't matter if you don't follow school rules as long as you get good scores. But if you are not good at this, you are just a good for nothing. I feel very disgusted with this”—P7.

#### School Refusal as a Strategy for Gaining Respite and Understanding

All 17 participants in the study stated that when the stress of academic competition became unbearable, they refused to go to school so that they could have a break and rest. In addition, most parents would have a dramatic change in their attitudes toward their children. They began to care about their children's feelings and needs instead of pushing them to learn.

“I told my parents that my school and the students around me were too good for me to catch up with. I wanted to take a year off. I could take a rest and make up for my lessons. Then, I could have a repeat year in school with less pressure”—P19.

“Now, my mom dares not to push me to finish all my homework or get high scores. She said she would accept each of my requirements as long as I go back to school. To be honest, I feel very relaxed, just go back to school sometimes to have fun with my classmates when I want to”—P3.

### Family Living Space Dominated by Conflicts

Our analysis indicated that from the adolescents' view, their family atmosphere was filled with criticism, unstable emotions, high expectations from their parents and marital conflicts between their parents.

#### Parents' Emotional Instability Inspiring Rebellious Behaviors

In addition to the teenagers, most parents were also influenced by career competition and survival stress. Many were unable to properly manage their expression of emotions in front of the adolescents. They became anxious, depressed or angry. Some parents were overcontrolling of their children. This would trigger more negative emotions of the adolescents, as well as rebellious behaviors such as school refusal. All 17 teenagers in the study emphasized the negative effects of their parents' emotional instability.

“It's my mom who needs psychotherapy. Her mood is very unstable; she will be super nervous if she finds me with a slightly poor score. Sometimes, she gets into a temper like a hysterical psychosis. Every time I see her like this, I feel very tired of living. I have to deal with my studies while dealing with her, which makes me more reluctant to study”—P8.

#### Inappropriate Family Expectations

In addition, 12 adolescents reported that their families had overly high expectations for their academic performance. For them, these expectations were far beyond their ability. When adolescents failed to meet expectations, their families criticized, judged or attacked them and continued to push them to study. This made them anxious, helpless, self-critical and angry. Many adolescents resisted satisfying the expectations of their families and refused to go to school.

“My father asked me to enter Fudan University, but this is far, far beyond my ability. I am not a ‘straight A student.’ I told him I could not do it, but he morally attacked me, saying I am not filial, not considerate of his hard work to make money. I just realized no matter how hard I tried, I couldn't make it, and he always felt bad. In this case, I'd rather not to go to school”—P7.

#### Imbalanced Parental Relationship

In some adolescents' families, there were unresolved marital conflicts between their parents, which led to dull, tense and unstable interpersonal relationships in the family. Many parents transferred their negative emotions caused by marital conflicts onto their children. The adolescents had to spend energy comforting their parents and responding to their emotions, which eliminated their enthusiasm for learning.

“My parents should never have gotten married. They have never had peace. They (my parents) quarrel almost every day; sometimes they even fight with each other. There was a time, as long as I was home, I got so annoyed, because I was always worried if they were going to fight. This distracted me from my homework and my papers”—P13.

“They (the parents) have been fighting with each other for many years. My dad does not care about my mom anymore. So, every time my mom gets frustrated, I have to take over my dad's responsibility to take care of his wife. To draw an analogy, if I have 100% energy, I have to spend at least 70% comforting my mom every day. I barely have the energy to deal with my studies”—P11.

From some teenagers' views, their parents translated their marital disappointment into high expectations of the children's academic performance, which made the adolescents anxious.

“My mother has always been disappointed with my father. She thinks she married the wrong man. She always nags me, ‘Study hard; don’t become a useless man like your father'; she has always pushed me to study. I feel like a scapegoat for her unhappy marriage, like the last hope for her life. When I think about all of this, I feel angry and anxious. It is not fair!”—P14.

#### Defect-Oriented Parenting Style

Many anxious parents focused on their children's shortcomings but rarely offered praise, appreciation or encouragement. This made the teenagers feel worthless and engage in self-denial. Therefore, they lacked confidence and motivation in dealing with learning challenges and were more likely to give up when frustrated in their studies. Our analysis suggested that most of these parents were dissatisfied with their own lives or their marriages and had high expectations of their adolescents' academic performance.

“My parents don't have self-recognition. Nor can they gain recognition from each other, which leads them to only see my flaws. Normally, they say other children are excellent but never praise me. After a while I was like, ‘Well, I’m crap; I can't make anything right, then what's the meaning of my studying?”'—P17.

#### School Refusal as a Buffer Against Family Conflicts

After the adolescents refused to go to school, family conflicts were temporarily relieved. It seemed that the school refusal forced the parents to acknowledge their family conflicts, took responsibility, and tried to solve them with different manners. Some parents temporarily stopped quarreling and tried to help their children through cooperative parental work. Some of them tried to control the excessive expression of their emotions. They had to lower their expectations of their children and began to praise their children. Consequently, some children were empowered and regained some confidence. In other words, many parents were forced to change because they had no other choice if they wanted their children to resume going to school.

“After I stopped going to school, my parents stopped quarreling and began to cooperate. They discuss how they can let me go back to school every day. Now we are much more ‘peaceful.’ Sometimes I think it's good, better than when they were fighting all the time before”—P13.

“My parents were much gentler with me after I got depressed and refuse to go to school. Especially my father, he now rarely loses his temper, has no requirements regarding my scores. I have a slight idea that I don't want to be recovered now actually, or they will make demands on me again”—P17.

### Personal Living Space Lacking Meaningful Support

The third superordinate theme explored the motivation for school refusal from the perspective of the adolescents' individual autonomy and existential experience.

#### An Arranged Life

Fifteen teenagers reported that their lives were excessively controlled by their families and teachers from an early age, including their living habits, time schedules, school choice, friend selection and hobbies. They did not have the right to manage their own lives. They lacked the opportunity to make their own plans and choices so that they did not experience the joy and satisfaction of growing, learning, and overcoming adversity.

“I feel like I've been living like a zombie since I was a baby. Everything has been arranged by my parents, including my education, so I feel like life has been boring since I was a child. I can almost see what I'll be like when I am 70 or 80 years old based on now. If everything is predetermined, why should I study so hard?”—P10.

For some adolescents, the experience of being excessively controlled in childhood made them even more resistant to their parents' demands, including to study at school.

“My life is like a precise instrument preprogrammed by my parents from the day I was born. I could not make any decisions about my life, from what clothes to wear and what food to eat, to what schools to enter and what friends to choose. Now I do not want to obey them anymore. I want to be myself”—P16.

#### Lack of Sense of Worth

Fourteen adolescents complained that long-term parental excessive control and negative judgment made their self-esteem extremely low. They felt they were neither worthy of living nor appreciated by others. Some adolescents did not believe they could be in charge of their own lives. As a result, they were more likely to give up when confronted with academic setbacks.

“To be honest, I do not feel I have any advantages really. I have no other skills to be proud of or any other life values. Getting high scores is my only ability to make my parents feel great. However, I failed this exam; I was deprived of the only capital that I had, good scores. I surrendered to life, didn't want to study anymore”—P15.

In some families, the reason why parents paid close attention to their children's shortcomings was closely related to the traditional Chinese culture, which advocates individual modesty. The adolescents' parents thought that even if their children have advantages, they should not give them too much praise; otherwise, it would make their children too proud to study hard.

“My dad always says, ‘Be humble.’ He is always worried that if he praises me, I'll self-aggrandize, so he never says anything nice about me. He always praises other children in front of me. After a time, I really felt very badly about myself that I was not good at anything compared to others”—P6.

#### An Empty Heart

Sixteen teenagers generally reported a strong feeling of emptiness in their hearts. Life was confusing and meaningless to them. They felt they had nothing to pursue, no expectations for life and no idea of the meaning to go to school.

“I was asked what I wanted to do with my life. To be honest, I really do not know what I want to do in the future; I do not even know what people are living for. My daily life is just going to school, doing exercises and taking exams. This kind of life… well, how to say, not painful, but very boring, a feeling that my heart is empty”—P2.

Eleven teenagers even claimed that studying was a meaningless job assigned to them by society and their families. They did not want to go to school. Nevertheless, they understood that school refusal was not accepted by society. This dilemma made them feel torn.

“Going to school was just a job assigned to me by the adults. I don't want to do it, but I can't give it up, or I'll be seen as a monster. Well… in fact, I study only to complete the task. Although I can get high scores and often get praise, my heart is always empty, feeling no sense of achievement”—P1.

#### School Refusal as a Challenge Against Authority for Freedom and Self-Development

Fourteen teenagers reported that school refusal became a way to revolt against parental authority. After they firmly refused to go to school, their parents' behavior pattern changed significantly, including their parents not overcontrolling their lives, starting to care about their feelings, and starting to praise them.

“Now I'm blackmailing my parents by not going to school, because I know that is what matters most to them. If I said I wouldn't go to school, my father would not dare to scold me, my mother would not dare to scream at me. They do not dare to come into my room without my permission. I'm free if I don't go to school”—P16.

Ten teenagers even developed new interests, hobbies and abilities during their absence from school. They made new friends through the internet, expanded their horizons, and improved their self-confidence.

“This semester, I didn't go to school; I found a new hobby, rap music. I record my own music with home devices and post it on Weibo and Bilibili. Many people give me thumbs-up and tell me that I have talent. I feel very happy, as I never have been recognized like this before”—P12.

“My favorite thing right now is to go to the comics club and draw comics with my friends and to do cosplay. Lots of people praise my painting or come to take photos with me, saying ‘Little sister, you are so good at cosplay.’ I feel very satisfied from my heart”—P18.

### Conflict Between the Pros and Cons of Being Labeled With a Psychiatric Diagnosis

Almost all teenagers in the study developed anxiety or depression due to school-related fatigue. Several were diagnosed by psychiatrists. The fourth superordinate theme captured the conflict between the pros and cons of being diagnosed with a mental disorder.

#### The Conflict Between “Should” and “Unwilling”

Fifteen teenagers reported that they were ambivalent about school. On the one hand, they thought they should go to school or they would not have a bright future. On the other hand, they were unwilling to study. They became trapped in such emotional dilemmas, becoming anxious, depressed and angry. Eight adolescents even met the diagnostic criteria for one type of mental disorder and claimed to be “mentally ill.”

“I'm on edge right now. I think I should go back to school, but it's too hard for me. But, I, I can't allow myself to give up school either. I'm so anxious, angry and helpless. The doctor said I had depression, and I agreed. I think I do have a ‘psychological problem”’—P17.

#### Being Bound by the Diagnosis and Emotions

Some adolescents considered themselves to be “psychotic patients.” They thought that psychiatric diagnoses such as depression prevented them from continuing school study. They believed that the precondition of resuming school was to eliminate “mental illness.”

“After the doctor diagnosed me with bipolar disorder, I understood why I couldn't go to school. It was not that I didn't want to go to school, but I couldn't. I need to recuperate, get more rest and adjust my emotions better”—P15.

#### The Benefits of “Psychological Problems”

Meanwhile, 10 teenagers reported that a diagnosis and emotional disturbance provided them with some “benefits,” that is, gave them a legitimate reason to stay out of school and thus avoid high expectations from families and intense academic competition from their peers. However, some teenagers had a sense of stigma and wished that others would not regard them as patients with mental disorders.

“To be honest, I dare not get well now, because if I recover from my illness, my parents will certainly revert to their high requirements of me. Hence, sometimes I think it's good to be a psychiatric patient”—P15.

“How to say it? The fact that I've been diagnosed with a mental illness is, in some ways, a good thing. Because my teachers and parents stopped pushing me to study after I got sick. But I also noticed that my classmates were looking at me strangely, as if I were a patient with a mental disorder. I don't like it”—P2.

### Reintegration in School Life

The last superordinate theme concerned the participants' experiences of returning to school. Eighteen adolescents in the study had attempted to return to school and experienced the following challenges and resources.

#### Returning to Studying

When adolescents tried to restart their studies after a period of school absenteeism, they faced fierce academic competition among classmates again and needed time to adjust to the learning intensity of the school. With their families' support, some adolescents in our study used their resources to develop resilience strategies to cope with the challenge. However, some failed and asked for suspension from school again.

“I tried several times to regain any course, but it didn't work. The pace of teaching in our school is too fast that if I just miss half a day, there will be a stack of papers waiting for me. I missed classes for such a long time that I couldn't keep up with my classmates now. I'm confused about this; I have no idea what to do”—P16.

“During the half year of rest time, my father helped me to find two tutors and a social training class to make up my English and math, and it's kind of worked out. Now I'm trying to go back to school, and I feel a bit relaxed in these two subjects”—P20.

#### Receiving Special Treatment

Nine teenagers reported that when they returned to school, some teachers and classmates treated them with special attitudes, including giving them excessive attention and inquiring about their situation. Some teenagers felt that they were treated as “abnormal” psychiatric patients. This made them uncomfortable, offended and frustrated. They wished they could be treated equally as the other students.

“When I went back to school a while ago, I was constantly being asked by my classmates, ‘What happened to you? Why have you been away from school for so long? Are you alright?’ I knew they meant no harm, but it just made me uncomfortable, feeling like I was being treated as a different monster”—P11.

“Our head teacher is very nervous now. Whenever she sees me with no smile on my face, she comes to me and asks if I am OK or do I need to see a doctor. It makes me very uncomfortable. I feel like she is paying too much attention to me, as I if I were a patient. Actually, all she needs to do is just treat me like the others”—P18.

#### Reconstructing Peer Belonging and the Meaning of Existing

Fifteen participants reported that after being absent from school for a time, they felt estranged from their classmates due to a lack of connection with them and lagging behind in their studies. This made them feel confused, lonely, and frustrated.

“Although, I wanted to go back to school now, I'm afraid to do so. Since I haven't seen my classmates for a long time, I feel like I don't have common topics with them. I don't understand their discussion about academic points either. Some students even tease me as if I were silly. I feel quite lonely, have nobody to talk to”—P11.

With assistance from their families and schools, some adolescents reconnected with their classmates using several strategies, such as talking with peers about common hobbies and participating in group activities in the class. Six participants reported that the above approaches began to take effect.

“To increase my contact with my classmates, my dad came up with the idea of inviting the boys in my class to visit his game design company together with me, and it worked! I finally had a chance to talk with those classmates I was unfamiliar with—P20.

“I think it's a virtuous circle. The more attached I am to the school, the more I want to participate in group activities, and then the more my classmates and teachers like me, and then, hmm… In fact, although I still dislike to do homework right now, I am willing to play with my classmates”—P20.

However, some participants failed to receive support from their families, peers and teachers. They still found themselves disagreeing with their classmates and had little sense of belonging. Then, they had to suspend their schooling again.

“I don't know, anyway… I've tried to talk to my classmates, but I just thought they were too immature to talk to. My parents seem to have given up on me. They hardly talk to me now, only take care of my younger brother. I don't want to go back to studying any more. Maybe I can start a small business in the future to feed myself, that's enough”—P5.

## Discussion

This is the first qualitative study to explore Chinese adolescents' experiences with school refusal. The first theme that emerged from the data concerned the Chinese social environment characterized by strong competition. Our results indicated that the anxiety and helplessness caused by high societal expectations were important factors that contributed to the adolescents' school refusal. This finding is consistent with reports from previous research showing that anxiety related to social expectations and school burnout were correlated with children's school absenteeism (8–10). Our analysis suggested that the emergence of this phenomenon may be related to fierce occupational competition and survival stress within the adult system. Parental anxiety about life might have been transmitted to their children. Moreover, it seems that the influence of parental anxiety on the children in this study may have existed since they were in kindergarten and have become more serious after they entered middle school (23). Meanwhile, the anxiety of adolescents and their families was reinforced and magnified by the teaching system. Finally, adolescents, parents and schools co-constructed an educational atmosphere that promoted anxiety.

Accordingly, school refusal may have unconsciously become a strategy adopted by the adolescents to obtain temporary relief from academic stress. This made their parents and teachers realize the adolescents' suffering and care more about the children' needs. They lowered their expectations of the children and stopped pushing them to study. Then the children's anxiety about academic performance was temporarily eased. This result partially coincides with previous findings that school refusal helps adolescents escape aversive social evaluative situations and supports them in pursuing attention from significant others (19, 20).

Regarding **the second theme**, *family living environment dominated by conflicts*, our analysis first suggested that parents' unstable emotions were closely related to adolescents' emotional stress such as anxiety. The latter may, in turn, have triggered or reinforced school refusal among the adolescents. This finding coincides with Bowen's systems theory that emotional stress among the parental subsystem is contagious in the family and might be transmitted to the children's subsystem through mechanism of low differentiation. Then, the children may develop emotional and behavioral problems including depression, anxiety and school refusal in response to the parents' unstable emotions (31). Meanwhile, it is partially consistent with the opinions of attachment theory that the emotional instability of caregivers is correlated with children's uncertainty and insecure attachment experience and related to more psychosomatic complaints among children (32). As implied by Li et al.'s (33) study, school refusers often display somatic symptoms including headache, abdominal pain, vomiting, nausea, dizziness, diarrhea, muscular ache, fatigue and palpitation, and anxiety was found to be the most recurrent etiology of those somatic complains. Moreover, prior studies have also suggested that children's school refusal is correlated with their interpersonal conflicts, their enmeshment with their parents and parental psychopathology (9, 13, 14, 16). Hence, a clinical implication of this finding is that psychotherapy for adolescents with school refusal may integrate interventions for the caregiver's emotional stress simultaneously.

Second, the findings that parents' marital conflicts contributed to adolescents' school refusal coincided with Bowen's triangulation theory (31). This result suggested that Chinese teenagers' school absenteeism may be triangulated with the marital conflicts of parents (34). The emergence of this subtheme also partially verified the conclusions of previous research that children involved in parental marital conflicts have poorer academic performance (35).

In terms of *inappropriate family expectations* and *defect-oriented parenting styles*, the findings on the former were consistent with Stierlin's delegation theory, which states that when family expectations are beyond the ability of the offspring, offspring may restrict their self-development and experience psychosomatic symptoms (36). Many parents in our study had overly high expectations for the adolescents' academic performance (high delegation). For the children, the delegation was overwhelming and made them anxious. It seems that they resisted satisfying their families' expectation, and develop school refusal as a challenge against the parental authority for freedom. Meanwhile, at the individual level, a defect-oriented parenting style may cause adolescents to *lack self-worth*, which makes them lack motivation to pursue academic achievements and have less confidence to deal with academic challenges (37).

Interestingly, we found that for the participants, school refusal acted as a buffer against family stress. The parents' attitudes toward their children changed, and their expectations decreased accordingly. This is partially consistent with the perspectives of systemic theory that symptoms such as school refusal may have functions including setting side family conflicts, controlling interpersonal relationships and expressing needs (38).

In the summary of the second theme, our analysis implied that the mechanisms reflected by the four subthemes not only jointly caused teenagers' school refusal but also each promoted the development of the other. In addition, these four subthemes may be linked with the first theme of *competition-oriented social living space*. For example, parents who were anxious about social competition and who were not satisfied with their marriages seemed more likely to delegate excessive expectations to their children and were more inclined to focus on the defects of their children. This may in turn have frustrated the learning motivation of teenagers.

Our third theme concerned the *lack of support for adolescents' personal living*. This theme reflected the youths' sense of emptiness in relation to the value and meaning of life. It has some similarity with the symptoms of *developmental depression*. Developmental depression has been proposed as sub-type of moderate depression that is a common and potentially normative developmental process of spiritual individuation in the pathway of late adolescent (39). The clinical presentation of developmental depression may include adolescents' frustration and anger in not finding meaning of their lives, a hunger for more connection and the struggle for existential value in a perceived valueless word (39). Our finding suggested that Chinese adolescents with school refusal might have also experienced the existential despair and struggle as their Western counterparts do during the process of developmental depression.

Meanwhile, our analysis implied that this phenomenon might be related to Chinese culture. Chinese tradition emphasizes filial piety and children's obedience to the family (40). In most families in our study, the adolescents might have experienced more excessive control from their families than their Western counterparts would experience (24). Additionally, Chinese culture emphasizes individual modesty. Thus, Chinese parents may be more reserved than Western parents in praising their children. Hence, the adolescents might have received less recognition from their families. These factors may together contribute to lower self-esteem, less intrinsic motivation and poorer mental resilience of adolescents (41). The adolescents might thus have become more vulnerable to academic stress and school absenteeism (11). Correspondingly, our analysis suggested that school refusal may have become a way for the adolescents to confront the authoritative education system and culture, balance their self-development with family expectations, and explore their new life goals.

The fourth theme regarded mental health diagnoses that both benefited and limited the adolescents. Our analysis verified the findings from previous research that mental health complaints such as depression were barriers preventing teenagers from going back to school (8, 14). Additionally, our analysis suggested that adolescents might have secondary gains from a psychiatric diagnosis. Mental health symptoms gave them a temporary respite from academic competition, overly high family expectations, and parental criticism. This is consistent with the symptomatic function emphasized in the theory of family therapy (38), suggesting that there may be some value in discussing the functions of psychiatric symptoms such as depression in psychotherapy for Chinese adolescents with school refusal.

The last factor concerned adolescents' experience of *reintegration into school*. The emergence of this theme implied that adolescents benefited from sincere acceptance from their teachers and classmates during their return to school. This finding coincides with the findings of previous research showing that a lack of interpersonal support from peers is an important contributor to children's school refusal (6, 14, 17, 42). Meanwhile, our analysis suggested that excessive attention from and special treatment from teachers and classmates hindered the adolescents' reintegration in school. Such treatment might be correlated with adolescents' stigma (43). It also implied that for adolescents with school refusal, a balance between being properly supported and being overprotected by people around them may be critical for their return to school.

In summary, our analysis emphasized the complex interacting effects of the social environment, family interpersonal conflicts, personal psychological factors and mental health complaints on the development and maintenance of adolescents' school refusal behaviors. These factors contributed to school refusal at each level. Meanwhile, these factors may interact and influence each other's effects on school refusal. This is consistent with EST (26, 27). In this study, teenagers who refused to go to school were placed at the center of their families, peers, school, and social contexts. The first ecological system, the microsystem, could include the adolescents' immediate interpersonal environmental surroundings with which they interacted, such as families and peers. The second layer was the meso-system. It could include the school and educational environments. The macrosystem could refer to the social and cultural environment characterized by high levels of anxiety in which all the other systems were nested. Our analysis implied that stress and dysfunctions occurring in the three systems co-contributed to adolescents' school refusal in an interactive manner. Therefore, interventions for Chinese teenagers with school refusal may need to integrate strategies that inspire reorganization and changes in different ecosystems, such as the release of new policies, improvement of peer relationships, restructuring of family systems and reconstruction of individual inner dynamics (39, 44).

### Limitations

There are several limitations to our study. The first limitation is that we only analyzed adolescents' experiences without interviewing their family members, such as their parents. This may have led to some deviation in the thematic structure achieved from our analysis. Future research could include the opinions of adolescents' families in the analysis and compare their experience with those of children. Second, some participants were diagnosed with mental health disorders. Their experience with school absenteeism might be different from those of adolescents without psychiatric diagnoses. Hence, it is suggested that future studies distinguish these two heterogeneous groups to obtain more specific conclusions. Third, although our findings implied some potential interventions for adolescents' school refusal, we did not focus on the exploration of effective treatment for school absenteeism. Future studies analyzing what children with school refusal identify as helpful in psychotherapy are suggested. Forth, it seems that some participants (e.g., P20) in our study had more supportive parents than the others. With a bigger sample, it might have been possible to describe different profiles of adolescents depending on their parents' parenting strategies (e.g., supported, pressurized, ignored, etc.), and the impact of these parenting strategies on the course of school refusal. Hence, future research related with this topic are also suggested. Fifth, although the current study took EST as the theoretical framework of reference, we also found other theories, such as Stierlin's family therapy and Bowen's systemic theory could be used to explain part of the themes and their interactions. However, the detailed connections between adolescents' school refusal and these theories have not been fully discussed. Thus, future studies analyzing the relationships of school refusal with other theoretical frameworks are strongly suggested.

## Data Availability Statement

The raw data supporting the conclusions of this article will be made available by the authors, without undue reservation.

## Ethics Statement

The studies involving human participants were reviewed and approved by the Ethics Committee of Tongji University as well as the Shanghai Pudong New Area Mental Health Center (No. PWRd2020-01). Written informed consent to participate in this study was provided by the participants' legal guardian/next of kin.

## Author Contributions

LL and XZ designed this study. LL, HG, and YW made substantial contributions to the participants' interviews, data transcriptions and manuscript draft, ensuring that the work was appropriately investigated and resolved. LL and YW conducted the data coding and analysis. All authors read and approved the final manuscript.

## Funding

This study received funding from the National Natural Science Foundation of China (Grant Number: 81771464), the Training Plan of Health System Academic Leader of the Shanghai Pudong Municipality Health Commission (Grant Number: PWRd2020-01).

## Conflict of Interest

The authors declare that the research was conducted in the absence of any commercial or financial relationships that could be construed as a potential conflict of interest.

## Publisher's Note

All claims expressed in this article are solely those of the authors and do not necessarily represent those of their affiliated organizations, or those of the publisher, the editors and the reviewers. Any product that may be evaluated in this article, or claim that may be made by its manufacturer, is not guaranteed or endorsed by the publisher.
